# The Role of Oxidative Stress-Induced Senescence in the Pathogenesis of Preeclampsia

**DOI:** 10.3390/antiox14050529

**Published:** 2025-04-28

**Authors:** Alexandra Barbouti, Dimitrios N. Varvarousis, Panagiotis Kanavaros

**Affiliations:** Department of Anatomy-Histology-Embryology, Faculty of Medicine, School of Health Sciences, University of Ioannina, 45110 Ioannina, Greece; dvarvarous@uoi.gr (D.N.V.); pkanavar@uoi.gr (P.K.)

**Keywords:** oxidative stress, cellular senescence, preeclampsia, placenta, ischemia and reperfusion

## Abstract

Preeclampsia is a hypertension condition of human pregnancy that poses a significant risk to pregnant women and their fetus. It complicates about 2–8% of human pregnancies worldwide and displays multifactorial pathogenesis, including increased placental oxidative stress because of disturbed utero-placental blood flow. Recent evidence suggests that increased oxidative stress promotes acceleration of the placental senescence which is implicated in the pathogenesis of preeclampsia. This review focuses on the mechanisms that lead to oxidative stress in preeclamptic patients and examines the role of oxidative stress-induced placental senescence in the pathogenesis of the disease.

## 1. Introduction

Preeclampsia typically occurs after twenty weeks of gestation; it is a severe multisystem disease of human pregnancy diagnosed by sudden-onset hypertension and at least one other associated complication, including proteinuria and dysfunction of the maternal organs [1,2,3,4]. It displays multifactorial pathogenesis including increased placental oxidative stress because of intermittent utero-placenta perfusion [1,2,3,5,6,7,8,9,10,11,12,13,14,15,16,17,18,19,20,21,22,23,24,25,26,27,28,29,30,31,32,33]. A growing body of evidence strongly suggests that increased oxidative stress promotes the acceleration of placental senescence, thereby contributing to the pathogenesis of preeclampsia [6,10,11,15,18,30,33].

Oxidative stress is a condition associated with the onset and progression of various diseases, including cardiovascular disease, ischemia and reperfusion syndrome, neurodegenerative diseases, cancer, as well as the physiological process of normal aging [34,35,36]. Oxidative stress results from an imbalance between the production and elimination of reactive species in cells and tissues. Reactive species are generated naturally in aerobic organisms and serve as physiological regulators in many essential cellular processes. However, when their levels exceed a critical threshold, they can react nonspecifically with biological macromolecules, leading to oxidation and disrupting redox homeostasis. At the cellular level, oxidative stress can result in cell death or trigger cellular senescence.

Cellular senescence is a state in which cells experience a prolonged and generally irreversible arrest of the cell cycle as a response to various stressful insults. This state is also characterized by increased levels of macromolecular damage, altered metabolism, and secretion of several factors, such as pro-inflammatory cytokines, growth factors, angiogenic factors, and matrix metalloproteinases, collectively called the senescence associated secretory phenotype (SASP) [37,38,39,40]. Senescence plays a vital role in maintaining tissue homeostasis as it prevents the proliferation of unwanted or damaged cells, thereby serving as an essential barrier against tumor development. However, senescent cells are not inactive; they can survive for extended periods and secrete factors that reinforce and spread senescence, contributing to tissue dysfunction. As a result, the accumulation of senescent cells can lead to several pathological conditions [37,38,39,40].

This review aims to uncover the key mechanisms driving oxidative stress in preeclamptic patients and to highlight the significant role of oxidative stress in triggering placental senescence. By thoroughly examining recent literature from leading scientific journals and online databases, we have selected articles that are relevant to our topic and demonstrate exceptional scientific rigor and influence in the field. Our findings are intended to enhance the understanding of the disease’s pathogenesis.

## 2. Definition and Types of Preeclampsia

**Figure 1:**
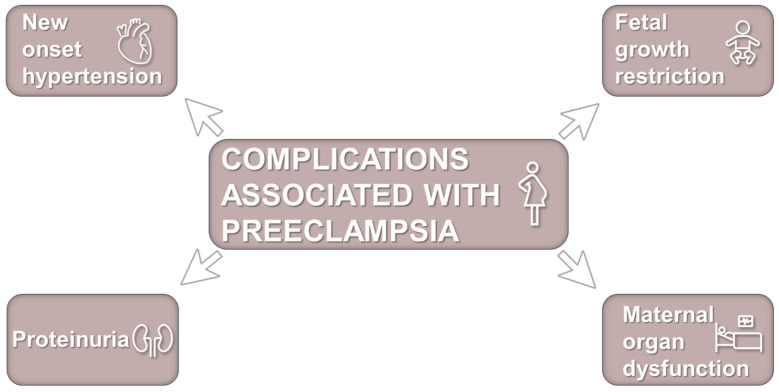
Complications associated with preeclampsia. Preeclampsia is an important cause of pregnancy complication that affects about 2–8% of human pregnancies worldwide and poses a significant risk to pregnant women and their fetus. It is characterized by maternal new onset hypertension observed after 20 weeks of gestation with (or without) proteinuria, other maternal organ dysfunction, and/or fetal growth restriction.

The current definition of preeclampsia from the International Society for the Study of Hypertension in Pregnancy comprises new onset hypertension (systolic >140 mmHg and diastolic >90 mmHg) after 20 weeks’ gestation accompanied by one or more other features: proteinuria, other maternal organ dysfunction (including liver, kidney, neurological), or hematological involvement, and/or uteroplacental dysfunction, such as fetal growth restriction and/or abnormal Doppler ultrasound findings of uteroplacental blood flow (Figure 1) [2]. Several risk factors have been recorded, such as chronic hypertension, autoimmune diseases such as systemic lupus erythematosus, chronic renal disease, nulliparity, previous stillbirth, pregestational diabetes high body mass index, maternal age, etc. [2,4,30]. Moreover, there is evidence indicating that gut microbiota alterations are implicated in the pathogenesis of preeclampsia [41].

There are two subtypes of preeclampsia: early- and the late-onset. The cut-off between the two types was set as before or after 34 weeks of gestation [8,30]. Early-onset preeclampsia is predominately due to defective placentation, including defective remodeling of the spiral arteries, during the first few weeks of pregnancy [8,30]. Late-onset preeclampsia, which represents about 80% of cases, is more likely to originate from a growing mismatch between the metabolic feto-placental demands and fetus and the maternal perfusion associated with a maternal genetic predisposition to cardiovascular disease [30].

Both types of preeclampsia cause syncytiotrophoblastic stress (e.g., oxidative, endoplasmic reticulum (ER), mitochondrial) and these cells exhibit features of increased senescence as a stress response [7,8,30]. As a result of the stress, the syncytiotrophoblasts release soluble factors, such as proinflammatory and anti-angiogenic mediators, into the maternal circulation, triggering endothelial dysfunction and a systemic inflammatory response leading to the clinical syndrome of preeclampsia [2,3,8,22,30]. Normal placental development is based on a branched vascular network and is dependent on various factors, such as vascular endothelial growth factor (VEGF), placental growth factor (PLGF), soluble fms-like tyrosine kinase-1 (sFlt-1), soluble endoglin (sEng), angiopoietin-1 (Ang-1), and angiopoietin-2 (Ang-2), which control and regulate normal blood vessel growth [2,3,8,22]. Imbalances in these regulatory factors can result in aberrant placental vascular development and contribute to the development of various pregnancy pathological conditions, including preeclampsia [1,2,3,8,22].

Moreover, there is evidence that preeclampsia is a state of chronic inflammation with activation of antigen presenting cells, T helper cells, B cells, and Natural killer cells which contribute to the symptoms during pregnancy [42]. For example, T helper cells secrete cytokines that induce the activation of innate immune cells and the production of anti-angiogenic factors [42]. Interestingly, PLGF is used in the prediction algorithm of preeclampsia in the first trimester, and in the second and third trimesters, the sFTL1/PLGF ratio is important for the prediction of preeclampsia, with a higher specificity than PLGF alone [43]. Furthermore, there is a body of evidence that some HTRA (High temperature requirement protease A) serine proteases including HTRA1, HTRA2, HTRA3 and HTRA4, which are normally expressed in placental villi, are impaired in pathological pregnancies such as preeclampsia, intrauterine growth restriction, and spontaneous preterm birth, suggesting that some of these proteins may be used as early markers of pregnancy outcome [44]. For example, a significant increase in HTRA1 plasma levels were found in pregnant women during 28–32 gestational weeks in cases of preeclampsia and preeclampsia- intrauterine growth restriction compared with the control group [44]. These are oligomeric serine proteases highly conserved from bacteria to humans and are involved in a variety of biological functions, including the maintenance of normal cell physiology and pathogenicity, such as cell growth, apoptosis, neurodegenerative disorders, inflammatory diseases and cancer [44]. These proteins are normally expressed in placental villi during all pregnancies, but their expression levels are altered in pathological pregnancies, suggesting a possible role of those proteins in the development of the human placenta [44]. Moreover, some HTRA family proteins have been found in maternal blood and were impaired in pathological pregnancy, suggesting a possible role of some of these proteins as early markers of pregnancy outcome [44].

## 3. Placental Development and Placenta Morphology in Preeclampsia

A summary of normal placental development is important to understand the pathophysiology of preeclampsia. The placenta is a feto-maternal organ that evades the maternal immune system to permit the survival of a semi-allogeneic fetus and maintains a healthy pregnancy by enabling the maternal–fetal exchange of nutrients and waste products [19,25,30].

The key placental cell line is the trophoblast cell line, which develops at the time of blastocyst formation and divides into two main cell subpopulations: (a) the villous trophoblasts that differentiate to villous cytotrophoblasts and the multinucleated villous syncytiotrophoblasts (derived from the fusion of cytotrophoblasts) forming the outer cover of all placental villi and (b) the extravillous trophoblasts (EVT) that are derived from the villous cytotrophoblasts and invade into the maternal uterine wall, reaching down to the inner third of the myometrium [7,19,30].

The EVT are formed at the areas where the anchoring tertiary villi has contact with the endometrium [7,19,30]. There is no covering syncytiotrophoblast at these sites, and the underlying villous cytotrophoblast proliferates and differentiates to EVT via a partial epithelial-mesenchymal transition (EMT), whereby polarized epithelial cells are transformed into invasive cells with partial mesenchymal phenotype [7,19,30,45]. EVTs characteristically express HLA-G and infiltrate the decidua migrating through the actions of secreted matrix metalloproteinases [30]. Huppertz [19] recently summarized the routes of EVT invasion in glands, veins, lymph vessels, and spiral arteries of the uterine wall. Briefly, (a) prior to six weeks of gestation, invasion of the early infiltrating syncytiotrophoblasts during implantation, as well as invasion of early EVT, results in the opening of the uterine glands and veins toward the placental intervillous space, (b) during this period the opening of the uterine glands by the infiltrating endoglandular EVT enables the flow of “uterine milk” (a mixture of cellular secretions, cellular debris, and transudation) toward the placenta (histotrophic nutrition), (c) later, during the first trimester of gestation, endoarterial EVT invade into uterine spiral arteries, transform their walls, and plug their lumen, thereby hindering flow of maternal blood into the placenta, and (d) at the beginning of the second trimester of gestation, the arterial EVT plugs gradually disintegrate, and the flow of maternal blood into the placenta is established (hemotrophic nutrition) [19].

The aforementioned model of normal placental development is disturbed in preeclampsia, and there is evidence that trophoblastic differentiation is diminished in the disorder [46,47]. Comparison of EVT of preeclamptic placentas with those of normal controls revealed multiple changes in gene expression profile, including changes indicative of a more limited EMT characterized by a more epithelial phenotype than the control EVT, suggesting that EVTs in preeclampsia display less invasive features, resulting in defective placentation [46]. Placental morphology is altered in preeclampsia. Macroscopic examination of placenta from preeclampsia reveals lesions that mainly reflect maternal malperfusion, including infarcts of the placenta tissue at different stages of resolution, retroplacental hemorrhage, and placental hypoplasia [5,8,30]. These lesions are not specific to the syndrome, and the spectrum of macroscopic lesions is more severe in early- compared with late-onset preeclampsia [30]. Microscopically various histological features have been reported in placenta tissue in preeclampsia, such as distal villous hypoplasia (scarcity of terminal villi with an obvious increase in intervillous space) and decidual arteriopathy that reflects inadequate spiral artery remodeling [5]. Decidual arteriopathy includes acute atherosis characterized by fibrinoid necrosis of the arterial wall, which is a dense, often thickened, eosinophilic material that is located beneath the endothelium and sometimes contains lipid-laden macrophages, incomplete or absent trophoblast invasion of the spiral artery wall, and arterial thrombosis [5].

## 4. Pathogenetic Implications of Oxidative Stress in Preeclampsia

**Figure 2:**
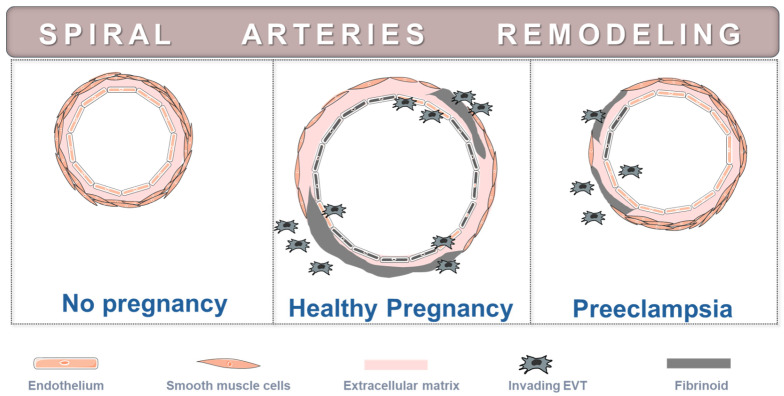
Diagram of decidual spiral artery remodeling in healthy pregnancy and in preeclampsia. Prior to remodeling, spiral arteries have an intact endothelium with extracellular matrix and a layer of vascular smooth muscle cells. In healthy pregnancy, extravillous trophoblasts (EVT) migrate and invade into decidual spiral arteries, transforming them into high-flow, low-resistance vessels and increasing the delivery of blood to the intervillous space. EVT invasion replaces the endothelium and destroys most of the musculo-elastic layer, which is replaced by fibrinoid material. In preeclampsia, EVT display less invasive features and fail to sufficiently transform the spiral arteries. The incomplete transformation involves the persistence of smooth muscle cells and lack of trophoblast invasion and fibrinoid deposition. Consequently, there is an intermittent perfusion of the intervillous space leading to repeated waves of hypoxia and reoxygenation.

There is evidence indicating that early-onset preeclampsia arises because of defective placentation that in turn increases the risk of oxidative stress [6,13,29,43,44]. The process of normal placentation is particularly intricate and involves (but is not restricted to) the coordinated migration and invasion of EVT into the maternal uterus and its luminal structures [46,47]. Invasion into the spiral arteries replaces the endothelium and destroys most of the musculo-elastic layer, which is replaced by amorphous fibrinoid material (Figure 2). This process transforms spiral arteries into wide diameter and low resistance vessels, enabling an adequate blood supply to the placenta. However, in preeclampsia, EVT display less invasive features and, hence, fail to sufficiently transform the spiral arteries (Figure 2). Defective transformation involves the persistence of smooth muscle cells and a deficient EVT invasion in a significant number of spiral arteries, resulting in intermittent perfusion and fluctuating oxygen concentrations within placental inter-villous space [7,8,14,30,46,47,48,49,50].

The repeated waves of hypoxia and reoxygenation provide the basis for an ischemia and reperfusion-type injury, which is mediated by several factors, including the elevated production of reactive oxygen species (ROS) [13,14,21,30,48]. The term ROS refers to a group of free radicals (e.g., superoxide (O_2_^•−^), hydroxyl radical (HO^•^)), and non-free radicals (e.g., hydrogen peroxide (H_2_O_2_)) that derive from the incomplete reduction of O_2_ and are more reactive than O_2_ itself [34,35,36]. Some ROS, such as the HO^•^ that is formed from H_2_O_2_ reduction in a Fe^2+^-catalyzed Fenton reaction, are extremely strong oxidizing agents [51]. Sustained accumulation of such highly reactive oxidants causes oxidative damage to biological macromolecules and gives rise to oxidative stress, a state that contributes to the onset and progression of numerous human diseases. Over the last three decades, oxidative stress received much attention as the major intermediary phenomenon in the generation of preeclampsia, although the underlying mechanisms are still under investigation.

In this section we begin with a brief overview of the global concept of oxidative stress. Next, we summarize the evidence implicating ROS in ischemia and reperfusion injury and the current understanding of the sources of ROS generation in post-ischemic tissues before moving on to a review of the evidence supporting the involvement of oxidative stress in the origination and development of preeclampsia.

### 4.1. The Dual Role of Reactive Species in Aerobes: Physiological Functions Versus Pathophysiological Consequences

**Figure 3:**
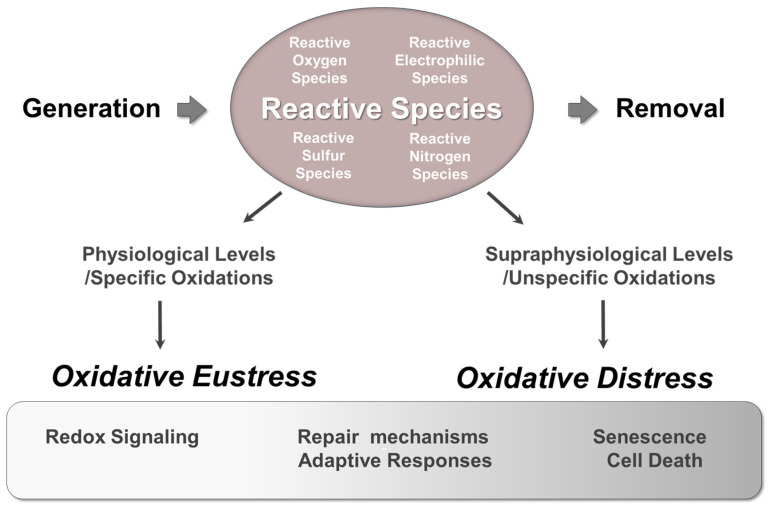
The dual role of reactive species in aerobes. The formation of several types of reactive species, including reactive oxygen species (ROS) (e.g., superoxide (O_2_^•−^), hydrogen peroxide (H_2_O_2_), hydroxyl radical (HO^•^), alkoxyl radical (RO^•^)) reactive nitrogen species (RNS) (e.g., nitric oxide (^•^NO), peroxynitrite (ONOO^−^)), reactive sulfur species (RSS) (e.g., hydrogen sulfide (H_2_S)), reactive electrophilic species (RES) (e.g., 4-hydroxy-2-nonenal (4-HNE), malondialdehyde (MDA), 4-oxo-2-nonenal (ONE)), is inevitable in aerobic cells. To prevent oxidative damage, several defense systems coordinate to attenuate them, contributing to the maintenance of a delicate redox balance. Reactive species play a dual role in aerobes: they are beneficial when confined to physiological levels (oxidative eustress), or damaging when their levels exceed a certain level (oxidative distress). They can also activate repair mechanisms or induce adaptive responses.

Oxidation-reduction (redox) reactions involve the transfer of electrons between chemical species. In these reactions, the species that loses electrons is said to be oxidized, while the species that gains electrons is reduced. Redox reactions are essential for life as they are intrinsically linked to cellular energy metabolism and regulate a wide range of essential cellular processes [34,52]. Various types of reactive species, such as reactive oxygen species (ROS), reactive nitrogen species (RNS), reactive sulfur species (RSS), and reactive electrophilic species (RES) are naturally generated in aerobic organisms through redox reactions (Figure 3) [36,53,54,55,56,57].

It is important to note that “reactive” is a relative term; reactive species include entities (free radicals and non-free radicals) with significantly different levels of chemical reactivity. Some reactive species are relatively unreactive and oxidize a limited number of cellular molecules, while others are highly reactive and can lead to uncontrolled oxidation of cellular components.

Cells respond to these species in various ways, depending on the nature, level, and duration of exposure as well as the cells’ ability to cope with the oxidants to which they are exposed [34,35,36]. To prevent oxidative damage to essential macromolecules, several defense systems coordinate to maintain a delicate balance between the generation and elimination of the diverse reactive species. When reactive species are confined to physiological concentrations and oxidize specific targets, they are not detrimental; instead, they play fundamentally important physiological roles as signaling entities that regulate vital cellular processes such as proliferation and differentiation. Additionally, cells typically respond to slight variation of the redox equilibrium by activating repair mechanisms or enhancing reducing defenses. This allows them to cope against oxidant insults and adapt to the new environmental conditions. However, when the levels of oxidants exceed a certain threshold, oxidative damage can accumulate and threaten cell integrity. Supraphysiological levels of oxidants may lead to senescence, a state of permanent growth arrest, or trigger various forms of cell death, including apoptosis, ferroptosis, necroptosis, and pyroptosis.

Consequently, reactive species function as pleiotropic agents, exhibiting multiple actions that can have either physiological functions or pathophysiological consequences. Lately, the terms, ’oxidative eustress’ and ‘oxidative distress’ have been introduced to distinguish between the beneficial effects and harmful impacts of oxidants in aerobic organisms. Eustress, derived from the Greek word “eu” meaning good, refers to the beneficial oxidative challenge, while oxidative distress refers to harmful effects resulting from supraphysiological oxidative challenge [58].

### 4.2. Ischemia and Reperfusion-Induced Injury

Ischemia refers to the partial or complete restriction of blood flow to specific areas of various tissues or entire organs. This condition leads to oxygen deficiency (hypoxia or anoxia), inadequate nutritional supply, and the accumulation of metabolic waste in the affected tissues. While cells can tolerate brief interruptions in blood supply without critical impairment in their functionality and viability, prolonged periods of ischemia lead to irreversibly injury. Therefore, the rapid restoration of perfusion is essential for salvaging the ischemic tissue and ensure survival.

Perhaps surprisingly, the reestablishment of blood flow to previously ischemic tissue can further exacerbate tissue damage and organ dysfunction, triggering a complex inflammatory response. In addition, in severe cases, exposure of a single organ to ischemia and reperfusion may elicit damage to remote organs, eventually leading to multiple organ dysfunction syndrome. This paradoxical aggravation of the injury upon reperfusion is known as ischemia and reperfusion injury, and it contributes to morbidity and mortality in various conditions [59,60,61,62]. Notably, it is strongly suggested that, in preeclampsia, the failure of physiological remodeling of the uteroplacental spiral arteries leads to intermittent perfusion of the intervillous tree, resulting in repeated cycles of hypoxia and reoxygenation [58,59,60,61]. This indicates that ischemia and reperfusion-type injury is one of the primary causes of the disease [1,63,64,65,66].

### 4.3. Reactive Oxygen Species as Contributors to Ischemia and Reperfusion Injury

Numerous mechanisms have been proposed to contribute to ischemia and reperfusion injury; however, there is growing evidence that a burst of ROS generation fueled by the re-introduction of oxygen to previously ischemic tissue, plays a critical role in the development of reperfusion injury. This understanding is largely derived from direct measurements of free radicals and evaluations of oxidative damage markers in post-ischemic tissues. Additionally, administering agents that reduce the initial burst of ROS generation during early reperfusion and implementing interventions that enhance ROS scavenging in experimental animal models exposed to ischemia and reperfusion have been shown to lessen the severity of reperfusion injury

Free radicals are typically evanescent and highly reactive, making them challenging to measure. The only method capable of directly and specifically detecting free radicals is electron spin resonance (ESR). In various organs subjected to ischemia and reperfusion, ESR spectroscopy and spin trapping techniques have revealed that O_2_^•−^ levels rise within seconds or minutes after O_2_ is re-introduced [67,68,69,70,71,72,73,74,75]. The formed O_2_^•−^ can then convert into other reactive species, like carbon-centered species or alkoxyl radicals [67,68].

An alternative to measure these reactive species directly is to detect the damage they cause (their cellular ‘fingerprints’). Increased oxidative damage to DNA, lipids, and proteins has frequently been observed in post-ischemic tissues [76,77]. Additional indirect evidence supporting the role of oxidative stress in this process comes from studies that report the protective effects of various antioxidant agents administrated before or during ischemia and reperfusion [62]. Recently, we demonstrated that combining membrane-permeable and impermeable iron-chelating drugs offers significant protection against ischemia and reperfusion injury. We propose that this approach neutralizes intracellular labile iron, thereby preventing the generation of highly reactive free radicals through the Fenton reaction and reducing subsequent tissue injury [78]. Finally, studies using animal models that either lack or overexpress ROS-scavenging enzymes further support the critical role of ROS in ischemia and reperfusion injury [79,80,81,82,83].

### 4.4. Sources and Biochemical Mechanisms of Reactive Oxygen Species Generation in Post-Ischemic Tissues

**Figure 4:**
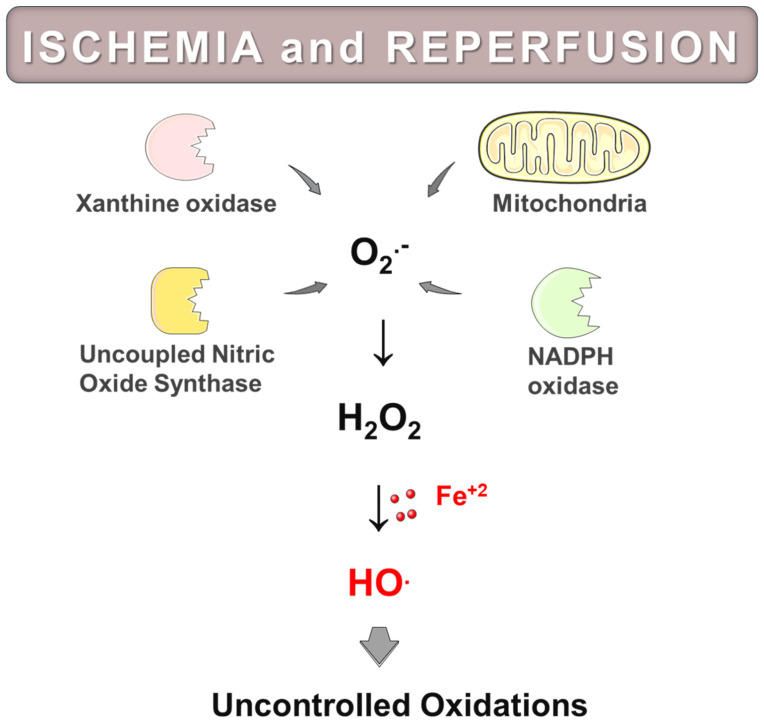
Sources of reactive oxygen species generation in post-ischemic tissues. Several different sources of reactive species generation have been proposed to contribute to reactive oxygen species generation when oxygen-starved tissues or organs are re-oxygenated. The most likely contributors are mitochondria, xanthine oxidase, NADPH oxidases, and uncoupled nitric oxide synthase. The generated O_2_^•−^ can be converted to downstream ROS, such as H_2_O_2_, which in turn produces HO^•^ via a reaction catalyzed by Fe^2+^ (Fenton reaction). HO^•^ is the most reactive oxidant produced in vivo and triggers unspecific oxidations to all biological molecules.

In recent decades, significant efforts have been made to understand the mechanisms that lead to excessive ROS production during reoxygenation of O_2_-starved tissues or organs. While various sources of ROS generation have been suggested, mitochondria, xanthine oxidase (XO), NADPH oxidases (NOX), and uncoupled nitric oxide synthase (NOS), are now recognized to be the most likely contributors (Figure 4).

XO is an enzyme expressed in many tissues that catalyzes the oxidation of hypoxanthine to xanthine and xanthine to uric acid while reducing molecular oxygen to O_2_^•−^. During prolonged periods of ischemia, xanthine dehydrogenase (XD) is converted to XO, and hypoxanthine accumulates due to the breakdown of ATP and insufficient washout from vascular spaces. Many authors have suggested that with the restoration of blood flow and tissue reoxygenation, XO generates increased amounts of O_2_^•−^, which in turn produce other downstream oxidants, such as H_2_O_2_. H_2_O_2_ can further react to form highly reactive HO^•^ through the Fenton reaction, contributing to reperfusion injury [60,61,62]. Furthermore, XO may play a role in systemic injury, such as multiple organ dysfunction syndrome. Significantly elevated XO levels have been detected in plasma after various organs experience ischemia and reperfusion. This suggests that circulating XO can initiate oxidative damage in organs that are located away from the initially affected area [60,61,62]. It is of interest to note that some studies indicate that ROS derived from XO may mediate ischemia and reperfusion injury by recruiting and activating neutrophils, rather than directly causing oxidative damage [84,85,86,87]. However, the role of XO in oxidative stress related to ischemia and reperfusion remains controversial. While many reports indicate a beneficial effect from inhibiting or deleting the enzyme prior to ischemia and reperfusion, some studies suggest that its contribution to the production of O_2_^•−^ is minimal [88,89,90]. In preeclamptic patients, elevated uric acid concentrations are commonly observed, which implies that XO might contribute to ROS generation and oxidative damage in this condition [91].

In addition to XO, the NOX family of enzymes have been proposed to mediate ischemia and reperfusion injury across various tissues [92,93,94]. Numerous studies conducted in different experimental models of ischemia and reperfusion have reported an upregulation of NOX expression and activity. Moreover, pharmacological or genetic inhibition of NOX activity has been shown to reduce ROS production and alleviate injury caused by ischemia and reperfusion. NOX2, the prototypical isoform expressed primarily in neutrophils, is quantitively the most significant NOX regarding O_2_^•−^ generation [95]. Ischemia and reperfusion prompt the release of ROS along with a range of proinflammatory mediators that recruit neutrophils to the surface of the vascular endothelium of the injured tissue. This recruitment is followed by the extravasation and migration of neutrophils into the tissues [96]. Once activated, neutrophils release elevated amounts of NOX2-derived reactive species, a process known as oxidative or respiratory burst. NOX2 transfers electrons from NADPH to O_2_ to generate O_2_^•−^, which rapidly produces H_2_O_2_. H_2_O_2_ is then converted to hypochlorous acid by the action of myeloperoxidase, or HO^•^, via the Fenton reaction. Both hypochlorous acid and HO^•^ are strong oxidants responsible for the antimicrobial action of neutrophils, but they can also cause collateral damage, contributing to tissue injury. Other NOX isoforms expressed in non-phagocytes, such as fibroblasts, vascular smooth muscle cells, and endothelial cells, produce lower levels of O_2_^•−^ and play essential roles as signaling entities [95]. However, evidence suggests that, during ischemia and reperfusion, these enzymes are upregulated by proinflammatory mediators and produce higher amounts of ROS, leading to oxidative damage in post-ischemic tissues [61]. In preeclampsia, the precise role of NOX enzymes remains unclear. Although several studies have indicated an increased expression or/and enzymatic activity of different NOX isoforms in endothelial cells or placental tissues from preeclampsia-affected women, other studies have not found significant differences when comparing normal and preeclamptic placentas, highlighting the need for further investigation [29,97,98,99,100,101].

NOS are a family of enzymes responsible for synthesizing ^•^NO, a gaseous free radical essential for various physiological processes, including vasodilation, neurotransmission, platelet aggregation and adhesion, and host defense [57]. In mammals, three isoforms have been identified: neuronal (nNOS), endothelial (eNOS) and inducible (iNOS) NOS. iNOS is primarily induced in immune cells and generates large quantities of ^•^NO over prolonged periods. When formed in excess, ^•^NO and other downstream nitrogen-derived reactive species can effectively kill microorganisms, playing crucial roles in host defense. In contrast, eNOS and nNOS generate lower to moderate amounts of ^•^NO, which have signaling properties. NOS enzymes possess the unique ability to switch their enzymatic activity and release O_2_^•−^ instead of ^•^NO. This phenomenon, known as NOS uncoupling, reduces ^•^NO bioavailability and exacerbates oxidative stress by potentiating ROS formation. Mechanistically, oxidative stress is a key contributor to NOS uncoupling, by degrading the substrate L-arginine, oxidizing the essential cofactor tetrahydrobiopterin (BH4), or inducing post-translational modifications to NOS. NOS uncoupling, particularly eNOS, has been observed in animal models and in patients experiencing ischemia and reperfusion. This process enhances O_2_^•−^ formation while decreasing ^•^NO bioavailability, further exacerbating oxidative damage in the reperfused tissue [102]. In the context of preeclampsia, significantly lower levels of L-arginine have been observed in preeclamptic compared to control placentas, suggesting that eNOS uncoupling may account for O_2_^•−^ formation in this disease [103,104]. Recently, Guerby et al., reported that eNOS is highly S-glutathionylated in preeclamptic placentas [105]. S-glutathionylation is the reversible binding of glutathione tripeptide to a protein, promoted by reactive oxygen or nitrogen species. This post-translational modification alters the structure, the folding, and the function of the modified protein. In the case of NOS, S-glutathionylation is one of the main mechanisms leading to enzyme uncoupling. Additionally, another recent study from the same group found that, in preeclamptic placentas, eNOS is modified by ONE, which is a reactive aldehyde generated during lipid peroxidation [106]. This modification leads to reduced ^•^NO generation in human trophoblastic cell lines, again indicating the loss of eNOS function.

While these enzymes are expressed in various tissues, their overall contribution to the generation of ROS during ischemia and reperfusion is believed to be less significant than that of mitochondria, and it tends to occur later in the process of ischemia and reperfusion injury [107]. This is not surprising since mitochondria represent a cornerstone of ROS generation in both physiological and pathological conditions. Most of the oxygen delivered to cells is consumed at the endpoint of the electron transport chain, that is, the cytochrome c oxidase (complex IV). This enzyme tends to tightly sequester electrons for the concerted four-electron reduction of O_2_, meaning it retains all partially reduced intermediates of O_2_ until it is safely reduced to water; therefore, it is not a source of ROS. However, the earlier components of the mitochondrial electron transport chain—namely complex I, III, and to some extent, complex II—generate O_2_^•−^ and, subsequently, other downstream ROS from O_2_ [108,109]. Under physiological conditions, these ROS are not damaging, but instead function as signaling molecules. However, during ischemia and reperfusion, as well as under other non-physiological conditions, mitochondria release elevated levels of ROS, contributing to oxidative stress.

During the ischemic period, little or no O_2_ is available to accept electrons, so they build up on the respiratory chain. For a long time, it was assumed that, upon reperfusion, the increased ROS production was the consequence of non-specific electron leakage from over-reduced mitochondrial electron transport chain components. This led to the view that ischemia and reperfusion injury was a random and chaotic series of damaging events [110,111]. However, a different perspective is now emerging, suggesting that ROS generation during reperfusion of previously ischemic tissue is a consequence of a widely conserved metabolic process. Murphy and colleagues showed that succinate, a citric acid cycle intermediate, accumulates markedly in diverse oxygen-starved organs due to the reverse action of succinate dehydrogenase (complex II), which reduces fumarate to succinate [111,112]. Upon the first minutes of reperfusion, the accumulated succinate is rapidly reoxidized by complex II, driving reverse electron transport at complex I, which generates a burst of O_2_^•−^. This generated O_2_^•−^, in turn, produces downstream ROS that provokes oxidative damage initiating ischemia and reperfusion injury [113]. According to this scenario, this mechanism contributes to O_2_^•−^ generation much more than any other source upon reperfusion [114].

A recent study provided evidence that in preeclamptic patients, and depending on the severity of the disease, dysfunctional mitochondria accumulate in the placenta [115]. Furthermore, mitochondrial respiration and H_2_O_2_ production were increased in healthy term placentas after in vitro exposure to hypoxia and reoxygenation [115]. Based on their findings, the authors suggested that ischemia and reperfusion increased mitochondrial respiration via a dysregulated electron transport chain and resulted in the increased levels of H_2_O_2_ in preeclamptic tissue [111]. In another study, morphological analysis using transmission electron microscopy revealed degenerative and apoptotic changes in mitochondria isolated from preeclamptic compared to control placentas [116]. Comparative proteomic analysis identified 26 mitochondrial proteins that were differentially expressed (four upregulated and 22 downregulated) in preeclamptic compared to normal placentas [116]. Bioinformatics analysis revealed that these proteins are involved in essential processes in the development of preeclampsia, for instance apoptosis, fatty acid oxidation, the respiratory chain, ROS generation, the tricarboxylic acid cycle and oxidative stress [116]. Furthermore, another recent study provided strong evidence of potential accumulation of unfolded or misfolded proteins coupled with impaired oxidative phosphorylation in mitochondria from preeclamptic patients, suggesting the involvement of increased ROS formation [13].

### 4.5. Damaging Effects of Oxidative Stress in Preeclampsia

Numerous studies have identified various oxidative stress markers, such as adducts, end products or oxidative modifications, which reflect reactions between reactive species and biological macromolecules in patients with preeclampsia. Reactive species can attack DNA, leading to multiple lesions, including strand breakage, abasic sites, and modifications to nucleotide bases [117]. Guanine is the base that is most susceptible to oxidation, and its oxidized form 8-hydroxy-2′-deoxyguanosine (8-OHdG) is a widely used marker for oxidative DNA damage. Immunohistochemical analysis of preeclamptic placentas showed that the proportion of 8-OHdG positive syncytiotrophoblast nuclei is significantly higher compared to control placentas, implying the involvement of oxidative stress in this pathology [17,18,28,31]. Notably, immunoreactivity for 8-OHdG was found to be higher in placentas of women with early-onset compared to late-onset preeclampsia [28].

Another major target of reactive species is the polyunsaturated fatty acids (PUFAs) chains in lipids [117]. Oxidation of PUFAs generates a wide variety of lipid peroxidation products, including reactive carbonyl compounds such as the MDA, HNE or ONE. These compounds exhibit strong electrophilic properties, allowing them to react spontaneously with numerous nucleophilic sites in proteins. This reaction forms protein adducts that progressively impair protein function [118,119,120]. Reactive carbonyls formed during lipid peroxidation along with their protein adducts, accumulate in oxidative stress-related diseases and aging, making them widely measured indicators of oxidative stress [121]. In preeclamptic pregnancies, both plasma and placental levels of MDA have been found to be significantly higher compared to normal pregnancies [122,123,124,125,126]. Furthermore, immunofluorescence and confocal microscopy studies have highlighted a strong accumulation of HNE-, acrolein-, and ONE-protein adducts in preeclamptic placentas, while their levels were low in placentas from normal pregnancies [106]. In addition, placental eNOS was modified by ONE in preeclamptic patients, but not in normal ones. This modification shifted eNOS enzymatic activity from a ^•^NO- to a O_2_^•−^-generating enzyme (a phenomenon referred to as NOS uncoupling) as indicated in human trophoblastic cell lines experiments [106]. Moreover, confocal imaging studies indicated the presence of HNE-adducts on sirtuin 1 (SIRT1) in preeclamptic placentas, whereas no such modifications were observed in placentas from normal pregnancies [127]. Since SIRT1 is a protein deacetylase, its carbonylation is expected to reduce its enzymatic activity. This is consistent with the accumulation of acetylated proteins seen in placental homogenates derived from preeclamptic pregnancies [127].

In addition to carbonyl-protein adducts, advanced oxidation protein products, which are oxidized aggregated proteins, also accumulate in plasma in various oxidative stress related diseases and ageing [128]. In the context of preeclampsia, there is evidence that women with high plasma levels of advanced oxidation protein products have a higher risk of developing this pathology [129], and their levels in plasma tend to increase with the severity of the disease [130]. Nevertheless, the precise role of advanced oxidation protein products remains to be illuminated as there are conflicting results in the literature [131].

Moreover, S-glutathionylated proteins also accumulate when redox balance switches in favor of reactive oxygen or nitrogen species. Recent reports indicate high S-glutathionylation for the eNOS enzyme in preeclamptic patients [105]. This modification is one of the main causes of NOS uncoupling. The switch of eNOS enzymatic activity towards O_2_^•−^ -formation exacerbates tissue damage by enhancing ROS formation and inactivating ^•^NO in preeclamptic placentas. It is likely, that S-glutathionylation of eNOS serves as both a cause and a consequence of increased reactive species formation in this disease.

Redox imbalances may lead to the accumulation of unfolded or misfolded proteins, disrupting cellular homeostasis. To compact or respond to protein unfolding or misfolding, cells have developed sophisticated stress response pathways known as unfolded protein responses (UPR). These pathways are found in all cellular compartments capable of protein synthesis and govern whether cells can re-establish homeostasis or initiate cell death programs [132]. Recent research has provided molecular evidence of the activation of ER and mitochondrial UPR mechanisms in placentas from cases of early-onset preeclampsia, suggesting the potential accumulation of unfolded or misfolded proteins in these organelles [12,13]. Notably, the activation of both pathways was largely restricted to the syncytiotrophoblast and endothelial cells. Similar observations were recapitulated in in vitro cellular models that mimic ischemia followed by reperfusion. Fluctuations in O_2_ levels (hypoxia and reoxygenation) in trophoblast-like cell lines showed a remarkable level of consistency in reproducing both the ER and the mitochondrial UPR signaling pathways of preeclamptic placentas [12]. In addition, the activation of mitochondrial UPR pathways causes impaired mitochondrial function, which increases the risk for further ROS formation [12,13]. There is also evidence of potential interactions between the ER and the mitochondrial UPR signaling pathways in early-onset preeclampsia [13].

The mitochondria and ER communicate and integrate their activities through membrane contact sites known as mitochondria-associated ER membranes (MAMs) [133]. The structure of MAMs is maintained by molecular bridges (protein tethers) that, among other crucial roles, modulate redox signaling between these organelles [134]. Recent studies showed that aquaporin11 (AQP11), a peroxiporin residing in the ER and accumulating partly in MAMs [135] transfers H_2_O_2_ from mitochondria to the ER [136]. Given the coupling of mitochondrial and ER function through MAMs, oxidative stress, mitochondrial stress and ER stress are closely interlinked, and it is unlikely that any one of these stresses occur in isolation [134,137,138,139]. Apparently, the redox interplay between these organelles determines the level of oxidative stress and its pathophysiological consequences in preeclampsia.

## 5. Pathogenetic Implications of Oxidative Stress-Induced Placental Senescence

Although some degree of oxidative stress occurs in normal pregnancies towards term, increased placental oxidative stress because of disturbed utero-placental blood flow and increased placental senescence resulting from various stressful stimuli, including oxidative stress are implicated in the pathogenesis of preeclampsia [6,7,10,11,15,18,30,33].

Placental senescence takes place in both normal and pathological pregnancies including preeclampsia [11,15,33,140,141,142,143,144]. Concerning normal pregnancy, the fusion of mature cytotrophoblasts to form the multinucleated syncytiotrophoblast layer during placentation is important for the placental and fetal development and has been recognized as a trigger of syncytiotrophoblast senescence which gradually progresses until term [8,15,140,141,142,143]. Indeed, Chuprin et al. [143] found in tissue sections that the third trimester normal syncytiotrophoblasts exhibit characteristics of cellular senescence such as (a) immunohistochemical expression of cyclin dependent kinase inhibitors (CDKI) p21 and p16, (b) immunohistochemical expression of the phosphorylated (serine-139) form of H2A histone family member X (γ-H2A.X) (a marker of double-strand DNA breaks), (c) absence of immunohistochemical expression of the cell proliferation-associated marker Ki67 and (d) senescence-associated beta-galactosidase (SA-βgal) enzymatic activity detected by histochemistry. This enzymatic activity, which is found in many normal cells under physiological conditions, is significantly elevated in senescent cells resulting from increased lysosomal content [40]. Thus, histochemical detection of SA-βgal activity at pH 6.0 (suboptimal for normal cells) permits specific identification of senescent cells [46]. Moreover, Chuprin et al. [143] showed in normal and carcinoma cell lines that expression of ERVWE1 (endogenous fusogenic protein) or infection by the fusogenic measles virus resulted in cell fusion and induction of a p53- or pRB-dependent senescence program. Moreover, Higuchi S. et al. [140] examined senescence-associated markers (SA-βgal, p16 and p21) in normal placenta and observed that (a) the histochemical expression of SA-βgal in cytotrophoblasts is strong in the first and second gestational trimesters, but weaker in the third trimester, (b) the syncytiotrophoblasts are SA-βgal negative in the first and second gestational trimesters, but become positive in the third trimester, and (c) the immunohistochemical expression of the CDKI p16 and p21 was stronger in cytotrophoblast than in syncytiotrophoblast throughout pregnancy, and the expression levels of these markers in syncytiotrophoblast were significantly increased as pregnancy advanced. In the same study, experiments using the BeWo human chorionic carcinoma cell line showed that the expression of SA-βgal, and p21 was stronger after than before cell fusion [140].

Concerning pathological pregnancies, including preeclampsia, the increase of placental senescence can be evidenced by the accumulation of senescence-associated markers, such as short telomeres, increased senescence-associated heterochromatin foci (SAHF), increased SASP phenotype, and elevated Sudan-Black-B histochemical staining, which detects lipofuscin granules (aggregates of oxidized lipids, proteins and metals known to accumulate in aged tissues), elevated enzymatic activity of SA-βgal, and increased immunohistochemical expression of phosphorylated (serine-139) histone γH2AX and the CDKI p16 and p21 [6,8,10,11,15,16,17,18,26,127,144,145,146]. This increase in placental senescence in preeclampsia can result from various mechanisms, such as oxidative stress, which may induce DNA-damage, accumulation of oxidatively damaged unfolded and/or misfolded proteins, mitochondrial dysfunction, and lipid peroxidation [7,8,9,10,11,14,15,17,18].

Τhe contribution of oxidative stress to the increase of senescence of trophoblastic cells was evidenced by in vitro studies using term human placenta explants and human cytotrophoblastic cell lines [6,8,24,127]. Indeed, oxidative stress induced by hypoxia and reoxygenation challenge resulted in senescent alterations in term placental explants in vitro, evidenced by (a) significant increase in the aggregation of lipofuscin granules, as detected by Sudan-Black-B histochemical staining, (b) significant increase in the expression levels of the CDKI p21 and p16, and (c) increased nuclear foci of modified histone γH2AX compared to normoxic controls [17]. Additionally, oxidative stress induced by H_2_O_2_ treatment caused DNA damage in term placental explants in vitro, evidenced by a higher proportion of nuclei showing immunohistochemical expression for 8-OHdG when compared to normoxic control explants [17]. In another study, treatment of the human trophoblast cell line HTR-8 with advanced oxidation protein products which are elevated in the plasma of preeclamptic pregnancies resulted in (a) increase of the levels of ROS, the levels of SA-βgal, the senescence-associated heterochromatin foci (SAHF) and the percentage of cells in the G0/G1 phase (indication of cell cycle arrest), and (b) decrease of cell mitochondrial membrane potential (ΔΨm) compared with the control group [23]. In the same study, (a) co-treatment of the trophoblastic cells with the antioxidant N-acetyl-L-cysteine (NAC) significantly reversed advanced oxidation protein products-induced senescence and (b) pre-treatment of the trophoblastic cells with rapamycin (an activator of autophagy) or 3-MA (an inhibitor of autophagy) significantly inhibited or promoted advanced oxidation protein products-induced senescence, respectively [23]. The authors concluded that advanced oxidation protein products may induce trophoblast cell senescence via oxidative stress and by inhibiting the autophagy process [23].

Further evidence for the oxidative stress-mediated senescence was provided by in vitro findings using lipid peroxidation-derived aldehydes [123]. As mentioned above, lipid peroxidation, which is caused by the oxidative attack of polyunsaturated fatty acids, generates various oxidation-derived molecules, including aldehydes such as HNE and ONE [147,148,149]. In this regard, the human trophoblastic cell line HTR-8 exhibited a significant increase in SA-βgal activity upon incubation with the lipid peroxidation-derived aldehydes HNE and ONE [6,127].

Elevated placental oxidative stress may also increase ER and mitochondrial stress, which contribute to the acceleration of placental senescence [14,15,150]. Indeed, as already mentioned, the accumulation of oxidatively damaged unfolded and/or misfolded proteins, which have potentially toxic cellular effects, may activate the ER and mitochondrial UPR pathways [12,13,21,150,151,152,153]. Interestingly, there is evidence that UPR participates in the induction of the senescent cell-cycle arrest pathway [154]. For example, stress-induced premature senescence can be promoted in proximal tubular epithelial cells treated in vitro with advanced glycation end-products which are protein glycosylation products involved in aging and age-related diseases via the activation of an ER stress-dependent p21 signaling [155]. Because of the coupling of mitochondrial and ER function through MAMs, oxidative, mitochondrial, and ER stress are interlinked and may have cooperative pathophysiological effects, such as acceleration of placental senescence in pregnancy complications, including preeclampsia [8,14,15,21,30]

Relevant to the above mentioned in vitro data [6,18,24,127] are histological findings in tissue sections that (a) trophoblastic cells in preeclamptic placentas show increased immunohistochemical expression of various oxidative stress markers (p.e., NOX, XO) and the oxidative DNA damage marker 8-OHdG in comparison to normal controls [17,18,23,26,27,28,31], and that (b) true syncytial knots (aggregates of aged and effete nuclei with highly condensed chromatin in the cytoplasm of syncytiotrophoblasts) are increased in preeclamptic placentas (Tenney–Parker change) and in advanced gestational age [156,157,158,159]. This increase of true syncytial knots has been attributed to premature placental aging, and immunohistochemical analysis of normal term and post-term placentas revealed that the true syncytial knots contained a high proportion of 8-OHdG-immunopositive nuclei, which also exhibit a highly condensed nuclear morphology (indication of aged and effete nuclei) in comparison to the 8-OHdG-immunonegative nuclei [151]. Moreover, placental explants exposed in vitro to H_2_O_2_ at concentrations that caused an increase in syncytial knot formation, showed significant increase in the percentage of 8-OHdG-immunopositive syncytiotrophoblastic nuclei in comparison to controls [157,158]. Taken together, the aforementioned in vitro and histological findings [6,18,127,157,158] support the concept that oxidative DNA damage contributes to the increased senescence in preeclamptic placentas.

## 6. Conclusions

The placenta is a feto-maternal organ that evades the maternal immune system, allowing the survival of a semi-allogeneic fetus and maintaining a healthy pregnancy by enabling the maternal–fetal exchange of nutrients and waste products. However, placental dysfunction is linked to several pregnancy complications, including preeclampsia and other ‘great obstetrical syndromes’. In preeclampsia, there is an increase in placental oxidative stress, which is caused by impaired utero-placental blood flow. This condition, along with the resulting placental senescence, is believed to play a significant role in the pathogenesis of the disease. Therefore, understanding the mechanisms that drive oxidative stress in preeclampsia and discovering ways to manipulate this condition is of major importance.

Strong evidence indicates that preeclampsia arises from defective placentation, which in turn leads to recurrent episodes of ischemia and reperfusion within the placental intervillous space. Although multiple factors contribute to the injury caused with the restoration of blood flow to previously ischemic tissues, a burst in the production of ROS plays a central role [13,14,21,30,48]. The term ROS refers to a diverse group of oxygen-derived free radicals and molecules with varying levels of reactivity [34,35,36]. For example, O_2_^•−^ and H_2_O_2_ are relatively weak oxidants, insufficient to cause direct damage to cellular macromolecules. In contrast, HO^•^ and RO^•^, which are generated via the Fe^2^⁺-catalyzed Fenton reaction from relatively unreactive peroxides are powerful oxidizing agents able to abstract electrons from any molecule that happens to be in the vicinity of their formation [51]. The ongoing accumulation of highly reactive oxidants leads to oxidative damage of biological macromolecules, resulting in oxidative stress.

Given that oxidative stress is associated with numerous human diseases, substantial efforts have been directed toward neutralizing highly reactive oxidants and mitigating their pathological impact. Traditionally, it has been assumed that dietary antioxidants—particularly free radical scavengers abundant in fruits and vegetables—could offer protection against oxidative stress and its associated diseases [160,161,162]. However, given the high reactivity and short lifespan of these species, it is unlikely that free radical scavenging antioxidants can neutralize them effectively in vivo [160,161,162]. Emerging evidence indicates that labile (redox-active) iron plays a pivotal role in ROS-mediated adverse effects [51]. Our previous studies have demonstrated that reducing intracellular iron levels can protect against oxidative stress-induced DNA damage and apoptotic cell death in various types of cells [161,163,164,165,166,167]. Furthermore, we have shown that pharmacological iron chelation effectively moderates liver ischemia-reperfusion injury in vivo [78]. These findings highlight the importance of targeting labile iron as a strategy to alleviate oxidative stress. Modulating intracellular iron availability may thus represent a promising and underexplored therapeutic approach for preventing or treating oxidative stress-related diseases.

## References

[1] Dimitriadis E., Rolnik D.L., Zhou W., Estrada-Gutierrez G., Koga K., Francisco R.P.V., Whitehead C., Hyett J., da Silva Costa F., Nicolaides K. (2023). Pre-Eclampsia. Nat. Rev. Dis. Primers.

[2] Brown M.A., Magee L.A., Kenny L.C., Karumanchi S.A., McCarthy F.P., Saito S., Hall D.R., Warren C.E., Adoyi G., Ishaku S. (2018). Hypertensive Disorders of Pregnancy: ISSHP Classification, Diagnosis, and Management Recommendations for International Practice. Hypertension.

[3] Ives C.W., Sinkey R., Rajapreyar I., Tita A.T.N., Oparil S. (2020). Preeclampsia—Pathophysiology and Clinical Presentations. J. Am. Coll. Cardiol..

[4] Erez O., Romero R., Jung E., Chaemsaithong P., Bosco M., Suksai M., Gotsch F. (2022). Preeclampsia and Eclampsia: The Conceptual Evolution of a Syndrome. Am. J. Obstet. Gynecol..

[5] Gusella A., Martignoni G., Giacometti C. (2024). Behind the Curtain of Abnormal Placentation in Pre-Eclampsia: From Molecular Mechanisms to Histological Hallmarks. Int. J. Mol. Sci..

[6] Negre-Salvayre A., Swiader A., Salvayre R., Guerby P. (2022). Oxidative Stress, Lipid Peroxidation and Premature Placental Senescence in Preeclampsia. Arch. Biochem. Biophys..

[7] Burton G.J., Cindrova-Davies T., Yung H.W., Jauniaux E. (2021). Oxygen and Development of the Human Placenta. Reproduction.

[8] Redman C.W.G., Staff A.C., Roberts J.M. (2022). Syncytiotrophoblast Stress in Preeclampsia: The Convergence Point for Multiple Pathways. Am. J. Obstet. Gynecol..

[9] Aouache R., Biquard L., Vaiman D., Miralles F. (2018). Oxidative Stress in Preeclampsia and Placental Diseases. Int. J. Mol. Sci..

[10] Manna S., McCarthy C., McCarthy F.P. (2019). Placental Ageing in Adverse Pregnancy Outcomes: Telomere Shortening, Cell Senescence, and Mitochondrial Dysfunction. Oxidative Med. Cell. Longev..

[11] Sultana Z., Maiti K., Dedman L., Smith R. (2018). Is There a Role for Placental Senescence in the Genesis of Obstetric Complications and Fetal Growth Restriction?. Am. J. Obstet. Gynecol..

[12] Yung H.W., Atkinson D., Campion-Smith T., Olovsson M., Charnock-Jones D.S., Burton G.J. (2014). Differential Activation of Placental Unfolded Protein Response Pathways Implies Heterogeneity in Causation of Early- and Late-Onset Pre-Eclampsia. J. Pathol..

[13] Yung H.W., Colleoni F., Dommett E., Cindrova-Davies T., Kingdom J., Murray A.J., Burton G.J. (2019). Noncanonical Mitochondrial Unfolded Protein Response Impairs Placental Oxidative Phosphorylation in Early-Onset Preeclampsia. Proc. Natl. Acad. Sci. USA.

[14] Burton G.J., Yung H.W., Cindrova-Davies T., Charnock-Jones D.S. (2009). Placental Endoplasmic Reticulum Stress and Oxidative Stress in the Pathophysiology of Unexplained Intrauterine Growth Restriction and Early Onset Preeclampsia. Placenta.

[15] Cox L.S., Redman C. (2017). The Role of Cellular Senescence in Ageing of the Placenta. Placenta.

[16] Farladansky-Gershnabel S., Gal H., Kidron D., Krizhanovsky V., Amiel A., Sukenik-Halevy R., Biron-Shental T. (2019). Telomere Homeostasis and Senescence Markers Are Differently Expressed in Placentas from Pregnancies with Early- Versus Late-Onset Preeclampsia. Reprod. Sci..

[17] Scaife P.J., Simpson A., Kurlak L.O., Briggs L.V., Gardner D.S., Pipkin F.B., Jones C.J.P., Mistry H.D. (2021). Increased Placental Cell Senescence and Oxidative Stress in Women with Pre-Eclampsia and Normotensive Post-Term Pregnancies. Int. J. Mol. Sci..

[18] Cindrova-Davies T., Fogarty N.M.E., Jones C.J.P., Kingdom J., Burton G.J. (2018). Evidence of Oxidative Stress-Induced Senescence in Mature, Post-Mature and Pathological Human Placentas. Placenta.

[19] Huppertz B. (2020). Traditional and New Routes of Trophoblast Invasion and Their Implications for Pregnancy Diseases. Int. J. Mol. Sci..

[20] Mizuuchi M., Cindrova-Davies T., Olovsson M., Charnock-Jones D.S., Burton G.J., Yung H.W. (2016). Placental Endoplasmic Reticulum Stress Negatively Regulates Transcription of Placental Growth Factor via ATF4 and ATF6β: Implications for the Pathophysiology of Human Pregnancy Complications. J. Pathol..

[21] Burton G.J., Yung H.W., Murray A.J. (2017). Mitochondrial—Endoplasmic Reticulum Interactions in the Trophoblast: Stress and Senescence. Placenta.

[22] Jung E., Romero R., Yeo L., Gomez-Lopez N., Chaemsaithong P., Jaovisidha A., Gotsch F., Erez O. (2022). The Etiology of Preeclampsia. Am. J. Obstet. Gynecol..

[23] Many A., Hubel C.A., Fisher S.J., Roberts J.M., Zhou Y. (2000). Invasive Cytotrophoblasts Manifest Evidence of Oxidative Stress in Preeclampsia. Am. J. Pathol..

[24] Li Z., Wang S., Li L. (2022). Advanced Oxidative Protein Products Drive Trophoblast Cells Into Senescence by Inhibiting the Autophagy: The Potential Implication of Preeclampsia. Front. Cell Dev. Biol..

[25] Burton G.J., Jauniaux E. (2017). The Cytotrophoblastic Shell and Complications of Pregnancy. Placenta.

[26] Londero A.P., Orsaria M., Marzinotto S., Grassi T., Fruscalzo A., Calcagno A., Bertozzi S., Nardini N., Stella E., Lellé R.J. (2016). Placental Aging and Oxidation Damage in a Tissue Micro-Array Model: An Immunohistochemistry Study. Histochem. Cell. Biol..

[27] Takagi Y., Nikaido T., Toki T., Kita N., Kanai M., Ashida T., Ohira S., Konishi I. (2004). Levels of Oxidative Stress and Redox-Related Molecules in the Placenta in Preeclampsia and Fetal Growth Restriction. Virchows Arch..

[28] Kimura C., Watanabe K., Iwasaki A., Mori T., Matsushita H., Shinohara K., Wakatsuki A. (2013). The Severity of Hypoxic Changes and Oxidative DNA Damage in the Placenta of Early-Onset Preeclamptic Women and Fetal Growth Restriction. J. Matern. Fetal Neonatal Med..

[29] Cui X.L., Brockman D., Campos B., Myatt L. (2006). Expression of NADPH Oxidase Isoform 1 (Nox1) in Human Placenta: Involvement in Preeclampsia. Placenta.

[30] Burton G.J., Redman C.W., Roberts J.M., Moffett A. (2019). Pre-Eclampsia: Pathophysiology and Clinical Implications. BMJ.

[31] Fujimaki A., Watanabe K., Mori T., Kimura C., Shinohara K., Wakatsuki A. (2011). Placental Oxidative DNA Damage and Its Repair in Preeclamptic Women with Fetal Growth Restriction. Placenta.

[32] Kohlrausch F.B., Keefe D.L. (2020). Telomere Erosion as a Placental Clock: From Placental Pathologies to Adverse Pregnancy Outcomes. Placenta.

[33] Sugulle M., Fiskå B.S., Jacobsen D.P., Fjeldstad H.E., Staff A.C. (2024). Placental Senescence and the Two-Stage Model of Preeclampsia. Am. J. Reprod. Immunol..

[34] Sies H., Belousov V.V., Chandel N.S., Davies M.J., Jones D.P., Mann G.E., Murphy M.P., Yamamoto M., Winterbourn C. (2022). Defining Roles of Specific Reactive Oxygen Species (ROS) in Cell Biology and Physiology. Nat. Rev. Mol. Cell. Biol..

[35] Sies H., Mailloux R.J., Jakob U. (2024). Fundamentals of Redox Regulation in Biology. Nat. Rev. Mol. Cell. Biol..

[36] Sies H., Jones D.P. (2020). Reactive Oxygen Species (ROS) as Pleiotropic Physiological Signalling Agents. Nat. Rev. Mol. Cell. Biol..

[37] Evangelou K., Belogiannis K., Papaspyropoulos A., Petty R., Gorgoulis V.G. (2023). Escape from Senescence: Molecular Basis and Therapeutic Ramifications. J. Pathol..

[38] Hernandez-Segura A., Nehme J., Demaria M. (2018). Hallmarks of Cellular Senescence. Trends Cell Biol..

[39] Gorgoulis V., Adams P.D., Alimonti A., Bennett D.C., Bischof O., Bishop C., Campisi J., Collado M., Evangelou K., Ferbeyre G. (2019). Cellular Senescence: Defining a Path Forward. Cell.

[40] Herranz N., Gil J. (2018). Mechanisms and Functions of Cellular Senescence. J. Clin. Investig..

[41] Zambella E., Peruffo B., Guarano A., Inversetti A., Di Simone N. (2024). The Hidden Relationship between Intestinal Microbiota and Immunological Modifications in Preeclampsia Pathogenesis. Int. J. Mol. Sci..

[42] Herrock O., Deer E., LaMarca B. (2023). Setting a Stage: Inflammation during Preeclampsia and Postpartum. Front. Physiol..

[43] Cristodoro M., Messa M., Tossetta G., Marzioni D., Dell’Avanzo M., Inversetti A., Di Simone N. (2024). First Trimester Placental Biomarkers for Pregnancy Outcomes. Int. J. Mol. Sci..

[44] Fantone S., Giannubilo S.R., Marzioni D., Tossetta G. (2021). HTRA Family Proteins in Pregnancy Outcome. Tissue Cell.

[45] Dasilva-Arnold S., James J.L., Al-Khan A., Zamudio S., Illsley N.P. (2015). Differentiation of First Trimester Cytotrophoblast to Extravillous Trophoblast Involves an Epithelial-Mesenchymal Transition. Placenta.

[46] Natenzon A., McFadden P., DaSilva-Arnold S.C., Zamudio S., Illsley N.P. (2022). Diminished Trophoblast Differentiation in Early Onset Preeclampsia. Placenta.

[47] Farah O., Nguyen C., Tekkatte C., Parast M.M. (2020). Trophoblast Lineage-Specific Differentiation and Associated Alterations in Preeclampsia and Fetal Growth Restriction. Placenta.

[48] Burton G.J., Jauniaux E. (2004). Placental Oxidative Stress: From Miscarriage to Preeclampsia. J. Soc. Gynecol. Investig..

[49] Jauniaux E., Hempstock J., Greenwold N., Burton G.J. (2003). Trophoblastic Oxidative Stress in Relation to Temporal and Regional Differences in Maternal Placental Blood Flow in Normal and Abnormal Early Pregnancies. Am. J. Pathol..

[50] Jauniaux E., Pahal G., Gervy C., Gulbis B. (2000). Blood Biochemistry and Endocrinology in the Human Fetus between 11 and 17 Weeks of Gestation. Reprod. Biomed. Online.

[51] Galaris D., Barbouti A., Pantopoulos K. (2019). Iron Homeostasis and Oxidative Stress: An Intimate Relationship. Biochim. Biophys. Acta (BBA)-Mol. Cell Res..

[52] Walsh C.T., Tu B.P., Tang Y. (2018). Eight kinetically stable but thermodynamically activated molecules that power cell metabolism. Chem. Rev..

[53] Radi R. (2018). Oxygen Radicals, Nitric Oxide, and Peroxynitrite: Redox Pathways in Molecular Medicine. Proc. Natl. Acad. Sci. USA.

[54] Lennicke C., Cochemé H.M. (2021). Redox Metabolism: ROS as Specific Molecular Regulators of Cell Signaling and Function. Mol. Cell..

[55] Parvez S., Long M.J.C., Poganik J.R., Aye Y. (2018). Redox Signaling by Reactive Electrophiles and Oxidants. Chem. Rev..

[56] Cirino G., Szabo C., Papapetropoulos A. (2023). Physiological roles of hydrogen sulfide in mammalian cells, tissues, and organs. Physiol. Rev..

[57] Lundberg J.O., Weitzberg E. (2022). Nitric Oxide Signaling in Health and Disease. Cell.

[58] Sies H., Berndt C., Jones D.P. (2017). Oxidative stress. Annu. Rev. Biochem..

[59] Eltzschig H.K., Eckle T. (2011). Ischemia and Reperfusion-from Mechanism to Translation. Nat. Med..

[60] Kalogeris T., Baines C.P., Krenz M., Korthuis R.J. (2017). Ischemia/Reperfusion. Compr. Physiol..

[61] Granger D.N., Kvietys P.R. (2015). Reperfusion Injury and Reactive Oxygen Species: The Evolution of a Concept. Redox Biol..

[62] Galaris D., Barbouti A., Korantzopoulos P. (2006). Oxidative Stress in Hepatic Ischemia-Reperfusion Injury: The Role of Antioxidants and Iron Chelating Compounds. Curr. Pharm. Des..

[63] Burton G.J., Woods A.W., Jauniaux E., Kingdom J.C.P. (2009). Rheological and Physiological Consequences of Conversion of the Maternal Spiral Arteries for Uteroplacental Blood Flow during Human Pregnancy. Placenta.

[64] Hung T.-H., Skepper J.N., Burton G.J. (2001). In Vitro Ischemia-Reperfusion Injury in Term Human Placenta as a Model for Oxidative Stress in Pathological Pregnancies. Am. J. Pathol..

[65] Jauniaux E., Poston L., Burton G.J. (2006). Placental-Related Diseases of Pregnancy: Involvement of Oxidative Stress and Implications in Human Evolution. Hum. Reprod. Update.

[66] Hung T.H., Burton G.J. (2006). Hypoxia and Reoxygenation: A Possible Mechanism for Placental Oxidative Stress in Preeclampsia. Taiwan J. Obstet. Gynecol..

[67] Raedschelders K., Ansley D.M., Chen D.D.Y. (2012). The Cellular and Molecular Origin of Reactive Oxygen Species Generation during Myocardial Ischemia and Reperfusion. Pharmacol. Ther..

[68] Zweierl J.L., Brodericko R., Kuppusamy P., Thompson-Gorman S., Luttyy G.A. (1994). Determination of the Mechanism of Free Radical in Human Aortic Endothelial Cells Exposed to Anoxia and Reoxygenation. J. Biol. Chem..

[69] Zweier J.L., Flaherty J.T., Weisfeldt M.L. (1987). Direct Measurement of Free Radical Generation Following Reperfusion of Ischemic Myocardium. Proc. Natl. Acad. Sci. USA.

[70] Garlick P.B., Davies M.J., Hearse D.J., Slater T.F. (1987). Direct Detection of Free Radicals in the Reperfused Rat Heart Using Electron Spin Resonance Spectroscopy. Circ. Res..

[71] Kadkhodaee M., Hanson G.R., Towner R.A., Endre Z.H. (1996). Detection of Hydroxyl and Carbon-Centred Radicals by EPR Spectroscopy after Ischaemia and Reperfusion of the Rat Kidney. Free Radic. Res..

[72] Miller C.W., Chen G., Janzen E.G. (1999). Detection of Free Radicals in Reperfused Dog Skin Flaps Using Electron Paramagnetic Resonance Spectroscopy: A Pilot Study. Microsurgery.

[73] Egemnazarov B., Sydykov A., Schermuly R.T., Weissmann N., Stasch J.-P., Sarybaev A.S., Seeger W., Grimminger F., Ghofrani H.A. (2009). Novel Soluble Guanylyl Cyclase Stimulator BAY 41-2272 Attenuates Ischemia-Reperfusion-Induced Lung Injury. Am. J. Physiol. Cell. Mol. Physiol..

[74] Togashi H., Shinzawa H., Matsuo T., Takeda Y., Takahashi T., Aoyama M., Oikawa K., Kamada H. (2000). Analysis of hepatic oxidative stress status by electron spin resonance spectroscopy and imaging. Free Radic. Biol. Med..

[75] Kono H., Woods C.G., Maki A., Connor H.D., Mason R.P., Rusyn I., Fujii H. (2006). Electron Spin Resonance and Spin Trapping Technique Provide Direct Evidence That Edaravone Prevents Acute Ischemia-Reperfusion Injury of the Liver by Limiting Free Radical-Mediated Tissue Damage. Free Radic. Res..

[76] Burke A., Fitzgerald G.A., Lucey M.R. (2002). A prospective analysis of oxidative stress and liver transplantation. Transplantation.

[77] Biasi F., Bosco M., Lanfranco G., Massano G., Donadio P.P., Vaj M., Andorno E., Rizzeti M., Salizzoni M., Poli G. (1995). Oxidative damage in human liver transplantation. Free Radic. Biol. Med..

[78] Mantelou A.G., Barbouti A., Goussia A., Zacharioudaki A., Papoudou-Bai A., Vlachou C., Kokkoris S., Papalois A., Galaris D., Glantzounis G.K. (2022). Combined Administration of Membrane-Permeable and Impermeable Iron-Chelating Drugs Attenuates Ischemia/Reperfusion-Induced Hepatic Injury. Free Radic. Biol. Med..

[79] Kinouchi H., Epsteintt C.J., Mizui T., Carlsont E., Chen S.F., Chan P.H. (1991). Attenuation of focal cerebral ischemic injury in transgenic mice overexpressing CuZn superoxide dismutase. Proc. Natl. Acad. Sci. USA.

[80] Horie Y., Wolf R., Flores S.C., Mccord J.M., Epstein C.J., Granger D.N. (1998). Transgenic mice with increased copper/zinc-superoxide dismutase activity are resistant to hepatic leukostasis and capillary no-reflow after gut ischemia/reperfusion. Circ. Res..

[81] Chen E., Bittner H., Davis R., Folz R., Van Trigt P. (1996). Extracellular Superoxide Dismutase Transgene Overexpression Preserves Postischemic Myocardial Function in Isolated Murine Hearts. Circulation.

[82] Li G., Chen Y., Saari J.T., Kangl Y.J., Kang Y.J. (1997). Catalase-overexpressing transgenic mouse heart is resistant to ischemia-reperfusion injury. Am. J. Physiol..

[83] Ishibashi N., Weisbrot-Lefkowitz M., Reuhl K., Inouye M., Mirochnitchenko O. (1999). Modulation of Chemokine Expression during Ischemia/Reperfusion in Transgenic Mice Overproducing Human Glutathione Peroxidases. J. Immunol..

[84] Meneshian A., Bulkley G.B. (2002). The Physiology of Endothelial Xanthine Oxidase: From Urate Catabolism to Reperfusion Injury to Inflammatory Signal Transduction. Microcirculation.

[85] Vega V., Mardones L., Maldonado M., Nicovani S., Manríquez V., Roa J., Ward P. (2000). Xanthine Oxidase Released from Reperfused Hind Limbs Mediate Kupffer Cell Activation, Neutrophil Sequestration, and Hepatic Oxidative Stress in Rats Subjected to Tourniquet Shock. Shock.

[86] Matsumura F., Yamaguchi Y., Goto M., Ichiguchi O., Akizuki E., Matsuda T., Okabe K., Liang J., Ohshiro H., Iwamoto T. (1998). Xanthine oxidase inhibition attenuates kupffer cell production of neutrophil chemoattractant following ischemia-reperfusion in rat liver. Hepatology.

[87] Otamiri T. (1989). Oxygen Radicals, Lipid Peroxidation, and Neutrophil Infiltration after Small-Intestinal Ischemia and Reperfusion. Surgery.

[88] Downey J.M., Miura T., Eddy L.J., Chambers D.E., Mellert T., Hearse D.J., Yellon D.M., Downey M., Miura T., Eddy L.J. (1987). Xanthine oxidase is not a source of free radicals in the ischemic rabbit heart. J. Mol. Cell. Cardiol..

[89] Kennedy T.P., Rao N.V., Hopkins C., Pennington L., Tolley E., Hoidal J.R. (1989). Role of Reactive Oxygen Species in Reperfusion Injury of the Rabbit Lung. J. Clin. Investig..

[90] Eddy L., Stewart J., Jones H., Engerson T., McCord J., Downey J. (1987). Free Radical-Producing Enzyme, Xanthine Oxidase, Is Undetectable in Human Hearts. Am. J. Physiol..

[91] Many A., Hubel C.A., Roberts J.M. (1996). Hyperuricemia and xanthine oxidase in preeclampsia, revisited. Am. J. Obstet. Gynecol..

[92] Lambeth J.D., Krause K.H., Clark R.A. (2008). NOX Enzymes as Novel Targets for Drug Development. Semin. Immunopathol..

[93] Kleikers P.W.M., Wingler K., Hermans J.J.R., Diebold I., Altenhöfer S., Radermacher K.A., Janssen B., Görlach A., Schmidt H.H.H.W. (2012). NADPH Oxidases as a Source of Oxidative Stress and Molecular Target in Ischemia/Reperfusion Injury. J. Mol. Med..

[94] Kahles T., Brandes R.P. (2013). Which NADPH Oxidase Isoform Is Relevant for Ischemic Stroke? The Case for Nox 2. Antioxid. Redox. Signal..

[95] Bedard K., Krause K.-H. (2007). The NOX Family of ROS-Generating NADPH Oxidases: Physiology and Pathophysiology. Physiol. Rev..

[96] Filippi M.D. (2019). Neutrophil Transendothelial Migration: Updates and New Perspectives. Blood.

[97] Myatt L., Cui X. (2004). Oxidative Stress in the Placenta. Histochem. Cell Biol..

[98] Chen J., Gao Q., Jiang L., Feng X., Zhu X., Fan X., Mao C., Xu Z. (2017). The NOX2-Derived Reactive Oxygen Species Damaged Endothelial Nitric Oxide System via Suppressed BKCa/SKCa in Preeclampsia. Hypertens. Res..

[99] Matsubara S., Sato I. (2001). Enzyme Histochemically Detectable NAD(P)H Oxidase in Human Placental Trophoblasts: Normal, Preeclamptic, and Fetal Growth Restriction-Complicated Pregnancy. Histochem. Cell Biol..

[100] Raijmakers M.T.M., Peters W.H.M., Steegers E.A.P., Poston L. (2004). NAD(P)H Oxidase Associated Superoxide Production in Human Placenta from Normotensive and Pre-Eclamptic Women. Placenta.

[101] Lim R., Acharya R., Delpachitra P., Hobson S., Sobey C.G., Drummond G.R., Wallace E.M. (2015). Activin and NADPH-Oxidase in Preeclampsia: Insights from in Vitro and Murine Studies. Am. J. Obstet. Gynecol..

[102] Roe N.D., Ren J. (2012). Nitric Oxide Synthase Uncoupling: A Therapeutic Target in Cardiovascular Diseases. Vascul. Pharmacol..

[103] Kim Y.J., Park H.S., Lee H.Y., Ha E.H., Suh S.H., Oh S.K., Yoo H.S. (2006). Reduced L-Arginine Level and Decreased Placental ENOS Activity in Preeclampsia. Placenta.

[104] Tashie W., Fondjo L.A., Owiredu W.K.B.A., Ephraim R.K.D., Asare L., Adu-Gyamfi E.A., Seidu L. (2020). Altered Bioavailability of Nitric Oxide and L-Arginine Is a Key Determinant of Endothelial Dysfunction in Preeclampsia. BioMed Res. Int..

[105] Guerby P., Swiader A., Augé N., Parant O., Vayssière C., Uchida K., Salvayre R., Negre-Salvayre A. (2019). High Glutathionylation of Placental Endothelial Nitric Oxide Synthase in Preeclampsia. Redox Biol..

[106] Guerby P., Swiader A., Tasta O., Pont F., Rodriguez F., Parant O., Vayssière C., Shibata T., Uchida K., Salvayre R. (2019). Modification of Endothelial Nitric Oxide Synthase by 4-Oxo-2(E)-Nonenal(ONE) in Preeclamptic Placentas. Free Radic. Biol. Med..

[107] Pell V.R., Chouchani E.T., Murphy M.P., Brookes P.S., Krieg T. (2016). Moving Forwards by Blocking Back-Flow the Yin and Yang of MI Therapy. Circ. Res..

[108] Brand M.D. (2016). Mitochondrial Generation of Superoxide and Hydrogen Peroxide as the Source of Mitochondrial Redox Signaling. Free Radic. Biol. Med..

[109] Bleier L., Wittig I., Heide H., Steger M., Brandt U., Dröse S. (2015). Generator-Specific Targets of Mitochondrial Reactive Oxygen Species. Free Radic. Biol. Med..

[110] Hausenloy D.J., Yellon D.M. (2013). Myocardial Ischemia-Reperfusion Injury: A Neglected Therapeutic Target. J. Clin. Investig..

[111] Murphy E., Steenbergen C. (2008). Mechanisms underlying acute protection from cardiac ischemia-reperfusion injury. Physiol. Rev..

[112] Chouchani E.T., Pell V.R., James A.M., Work L.M., Saeb-Parsy K., Frezza C., Krieg T., Murphy M.P. (2016). A Unifying Mechanism for Mitochondrial Superoxide Production during Ischemia-Reperfusion Injury. Cell Metab..

[113] Sorby-Adams A., Prime T.A., Miljkovic J.L., Prag H.A., Krieg T., Murphy M.P. (2024). A Model of Mitochondrial Superoxide Production during Ischaemia-Reperfusion Injury for Therapeutic Development and Mechanistic Understanding. Redox Biol..

[114] Murphy M.P. (2009). How Mitochondria Produce Reactive Oxygen Species. Biochem. J..

[115] Holland O.J., Cuffe J.S.M., Dekker Nitert M., Callaway L., Kwan Cheung K.A., Radenkovic F., Perkins A.V. (2018). Placental Mitochondrial Adaptations in Preeclampsia Associated with Progression to Term Delivery. Cell Death Dis..

[116] Shi Z., Long W., Zhao C., Guo X., Shen R., Ding H. (2013). Comparative Proteomics Analysis Suggests That Placental Mitochondria Are Involved in the Development of Pre-Eclampsia. PLoS ONE.

[117] Murphy M.P., Bayir H., Belousov V., Chang C.J., Davies K.J.A., Davies M.J., Dick T.P., Finkel T., Forman H.J., Janssen-Heininger Y. (2022). Guidelines for Measuring Reactive Oxygen Species and Oxidative Damage in Cells and in Vivo. Nat. Metab..

[118] Esterbauer H., Schaur R.J., Zollner H. (1991). Chemistry and biochemistry of 4-hydroxynonenal, malonaldehyde and related aldehydes. Free Radic. Biol. Med..

[119] Forman H.J., Fukuto J.M., Miller T., Zhang H., Rinna A., Levy S. (2008). The Chemistry of Cell Signaling by Reactive Oxygen and Nitrogen Species and 4-Hydroxynonenal. Arch. Biochem. Biophys..

[120] Negre-Salvayre A., Coatrieux C., Ingueneau C., Salvayre R. (2008). Advanced Lipid Peroxidation End Products in Oxidative Damage to Proteins. Potential Role in Diseases and Therapeutic Prospects for the Inhibitors. Br. J. Pharmacol..

[121] Benedetti A., Comportia M., Esterbauerb H. (1980). Identification of 4-hydroxynonenal as a cytotoxic product originating from the peroxidation of liver microsomal lipids. Biochim. Biophys. Acta..

[122] Madazli R., Benian A., Aydin S., Uzun H., Tolun N. (2002). The Plasma and Placental Levels of Malondialdehyde, Glutathione and Superoxide Dismutase in Pre-Eclampsia. J. Obstet. Gynaecol..

[123] Afrose D., Chen H., Ranashinghe A., Liu C.-c., Henessy A., Hansbro P.M., McClements L. (2022). The Diagnostic Potential of Oxidative Stress Biomarkers for Preeclampsia: Systematic Review and Meta-Analysis. Biol. Sex Differ..

[124] Sahay A.S., Sundrani D.P., Wagh G.N., Mehendale S.S., Joshi S.R. (2015). Regional Differences in the Placental Levels of Oxidative Stress Markers in Pre-Eclampsia. Int. J. Gynaecol. Obstet..

[125] Aydin S., Benian A., Madazli R., Uludaǧ S., Uzun H., Kaya S. (2004). Plasma Malondialdehyde, Superoxide Dismutase, SE-Selectin, Fibronectin, Endothelin-1 and Nitric Oxide Levels in Women with Preeclampsia. Eur. J. Obstet. Gynecol. Reprod. Biol..

[126] Kaur G., Mishra S., Sehgal A., Prasad R. (2008). Alterations in Lipid Peroxidation and Antioxidant Status in Pregnancy with Preeclampsia. Mol. Cell. Biochem..

[127] Tasta O., Swiader A., Grazide M.H., Rouahi M., Parant O., Vayssière C., Bujold E., Salvayre R., Guerby P., Negre-Salvayre A. (2021). A Role for 4-Hydroxy-2-Nonenal in Premature Placental Senescence in Preeclampsia and Intrauterine Growth Restriction. Free Radic. Biol. Med..

[128] Witko-Sarsat V., Friedlander M., Capeillere-Blandin C., Nguyen-Khoa T., Nguyen A.T., Zingraff J., Jungers P., Descamps-Latscha B. (1996). Advanced oxidation protein products as a novel marker of oxidative stress in uremia. Kidney Int..

[129] Karacay Ö., Sepici-Dincel A., Karcaaltincaba D., Sahin D., Yalvaç S., Akyol M., Kandemir Ö., Altan N. (2010). A Quantitative Evaluation of Total Antioxidant Status and Oxidative Stress Markers in Preeclampsia and Gestational Diabetic Patients in 24-36 Weeks of Gestation. Diabetes Res. Clin. Pract..

[130] Huang Q.T., Zhong M., Tian J.W., Hou F.F. (2013). Higher Plasma AOPP Is Associated with Increased Proteinuria Excretion and Decreased Glomerular Filtration Rate in Pre-Eclamptic Women. Pregnancy Hypertens..

[131] Noyan T., Güler A., Şekeroǧlu M.R., Kamaci M. (2006). Serum Advanced Oxidation Protein Products, Myeloperoxidase and Ascorbic Acid in Pre-Eclampsia and Eclampsia. Aust. N. Z. J. Obstet. Gynaecol..

[132] Lindholm D., Korhonen L., Eriksson O., Kυks S. (2017). Recent Insights into the Role of Unfolded Protein Response in ER Stress in Health and Disease. Front. Cell. Dev. Biol..

[133] Prinz W.A., Toulmay A., Balla T. (2020). The Functional Universe of Membrane Contact Sites. Nat. Rev. Mol. Cell. Biol..

[134] Resende R., Fernandes T., Pereira A., Marques A., Pereira C. (2022). Endoplasmic Reticulum-Mitochondria Contacts Modulate Reactive Oxygen Species-Mediated Signaling and Oxidative Stress in Brain Disorders: The Key Role of Sigma-1 Receptor. Antiox. Red. Signal..

[135] Bestetti S., Galli M., Sorrentino I., Pinton P., Rimessi A., Sitia R., Medraño-Fernandez I. (2020). Human Aquaporin-11 Guarantees Efficient Transport of H_2_O_2_ across the Endoplasmic Reticulum Membrane. Redox Biol..

[136] Sorrentino I., Galli M., Medraño-Fernandez I., Sitia R. (2022). Transfer of H_2_O_2_ from Mitochondria to the Endoplasmic Reticulum via Aquaporin-11. Redox Biol..

[137] Senft D., Ronai Z.A. (2015). UPR, Autophagy, and Mitochondria Crosstalk Underlies the ER Stress Response. Trends Biochem. Sci..

[138] Galluzzi L., Kepp O., Trojel-Hansen C., Kroemer G. (2012). Mitochondrial Control of Cellular Life, Stress, and Death. Circ. Res..

[139] Yoboue E.D., Sitia R., Simmen T. (2018). Redox Crosstalk at Endoplasmic Reticulum (ER) Membrane Contact Sites (MCS) Uses Toxic Waste to Deliver Messages. Cell Death Dis..

[140] Higuchi S., Miyamoto T., Kobara H., Yamada S., Asaka R., Kikuchi N., Kashima H., Ohira S., Shiozawa T. (2019). Trophoblast Type-Specific Expression of Senescence Markers in the Human Placenta. Placenta.

[141] Gauster M., Moser G., Wernitznig S., Kupper N., Huppertz B. (2022). Early Human Trophoblast Development: From Morphology to Function. Cell. Mol. Life Sci..

[142] Goldman-Wohl D., Yagel S. (2014). United We Stand Not Dividing: The Syncytiotrophoblast and Cell Senescence. Placenta.

[143] Chuprin A., Gal H., Biron-Shental T., Biran A., Amiel A., Rozenblatt S., Krizhanovsky V. (2013). Cell Fusion Induced by ERVWE1 or Measles Virus Causes Cellular Senescence. Genes Dev..

[144] Farfαn-Labonne B., Leff-Gelman P., Pellσn-Dνaz G., Camacho-Arroyo I. (2023). Cellular Senescence in Normal and Adverse Pregnancy. Reprod. Biol..

[145] Biron-Shental T., Sukenik-Halevy R., Sharon Y., Goldberg-Bittman L., Kidron D., Fejgin M.D., Amiel A. (2010). Short Telomeres May Play a Role in Placental Dysfunction in Preeclampsia and Intrauterine Growth Restriction. Am. J. Obstet. Gynecol..

[146] Sukenik-Halevy R., Amiel A., Kidron D., Liberman M., Ganor-Paz Y., Biron-Shental T. (2016). Telomere homeostasis in trophoblasts and in cord blood cells from pregnancies complicated with preeclampsia. Am. J. Obstet. Gynecol..

[147] Higdon A., Diers A.R., Oh J.Y., Landar A., Darley-Usmar V.M. (2012). Cell Signalling by Reactive Lipid Species: New Concepts and Molecular Mechanisms. Biochem. J..

[148] Castro J.P., Jung T., Grune T., Siems W. (2017). 4-Hydroxynonenal (HNE) Modified Proteins in Metabolic Diseases. Free Radic. Biol. Med..

[149] Chapple S.J., Cheng X., Mann G.E. (2013). Effects of 4-Hydroxynonenal on Vascular Endothelial and Smooth Muscle Cell Redox Signaling and Function in Health and Disease. Redox Biol..

[150] Correia-Melo C., Passos J.F. (2015). Mitochondria: Are They Causal Players in Cellular Senescence?. Biochim. Biophys. Acta (BBA)-Bioenerg..

[151] Holland O., Dekker Nitert M., Gallo L.A., Vejzovic M., Fisher J.J., Perkins A.V. (2017). Review: Placental Mitochondrial Function and Structure in Gestational Disorders. Placenta.

[152] Jovaisaite V., Mouchiroud L., Auwerx J. (2014). The Mitochondrial Unfolded Protein Response, a Conserved Stress Response Pathway with Implications in Health and Disease. J. Exp. Biol..

[153] Mukherjee I., Dhar R., Singh S., Sharma J.B., Nag T.C., Mridha A.R., Jaiswal P., Biswas S., Karmakar S. (2021). Oxidative Stress-Induced Impairment of Trophoblast Function Causes Preeclampsia through the Unfolded Protein Response Pathway. Sci. Rep..

[154] Pluquet O., Pourtier A., Abbadie C. (2015). The Unfolded Protein Response and Cellular Senescence. A Review in the Theme: Cellular Mechanisms of Endoplasmic Reticulum Stress Signaling in Health and Disease. Am. J. Physiol. Cell Physiol..

[155] Liu J., Huang K., Cai G.Y., Chen X.M., Yang J.R., Lin L.R., Yang J., Huo B.G., Zhan J., He Y.N. (2014). Receptor for Advanced Glycation End-Products Promotes Premature Senescence of Proximal Tubular Epithelial Cells via Activation of Endoplasmic Reticulum Stress-Dependent P21 Signaling. Cell Signal..

[156] Calvert S.J., Jones C.J.P., Sibley C.P., Aplin J.D., Heazell A.E.P. (2013). Analysis of Syncytial Nuclear Aggregates in Preeclampsia Shows Increased Sectioning Artefacts and Decreased Inter-Villous Bridges Compared to Healthy Placentas. Placenta.

[157] Fogarty N.M.E., Ferguson-Smith A.C., Burton G.J. (2013). Syncytial Knots (Tenney-Parker Changes) in the Human Placenta: Evidence of Loss of Transcriptional Activity and Oxidative Damage. Am. J. Pathol..

[158] Heazell A.E.P., Moll S.J., Jones C.J.P., Baker P.N., Crocker I.P. (2007). Formation of Syncytial Knots Is Increased by Hyperoxia, Hypoxia and Reactive Oxygen Species. Placenta.

[159] Donthi D., Malik P., Mohamed A., Kousar A., Subramanian R.A., Manikyam U.K. (2020). An Objective Histopathological Scoring System for Placental Pathology in Pre-Eclampsia and Eclampsia. Cureus.

[160] Barbouti A., Goulas V. (2021). Dietary Antioxidants in the Mediterranean Diet. Antioxidants.

[161] Barbouti A., Lagopati N., Veroutis D., Goulas V., Evangelou K., Kanavaros P., Gorgoulis V.G., Galaris D. (2021). Implication of Dietary Iron-Chelating Bioactive Compounds in Molecular Mechanisms of Oxidative Stress-Induced Cell Ageing. Antioxidants.

[162] Barbouti A., Briasoulis E., Galaris D. (2010). Protective Effects of Olive Oil Components Against Hydrogen Peroxide-Induced DNA Damage. Olives and Olive Oil in Health and Disease Prevention.

[163] Nousis L., Kanavaros P., Barbouti A. (2023). Oxidative Stress-Induced Cellular Senescence: Is Labile Iron the Connecting Link?. Antioxidants.

[164] Barbouti A., Amorgianiotis C., Kolettas E., Kanavaros P., Galaris D. (2007). Hydrogen Peroxide Inhibits Caspase-Dependent Apoptosis by Inactivating Procaspase-9 in an Iron-Dependent Manner. Free. Radic. Biol. Med..

[165] Tenopoulou M., Doulias P.-T., Barbouti A., Brunk U., Galaris D. (2005). Role of Compartmentalized Redox-Active Iron in Hydrogen Peroxide-Induced DNA Damage and Apoptosis. Biochem. J..

[166] Barbouti A., Doulias P.-T., Nousis L., Tenopoulou M., Galaris D. (2002). DNA Damage and Apoptosis in Hydrogen Peroxide-Exposed Jurkat Cells: Bolus Addition versus Continuous Generation of H_2_O_2_. Free. Radic. Biol. Med..

[167] Barbouti A., Doulias P.-T., Zhu B.-Z., Frei B., Galaris D. (2001). Intracellular Iron, but Not Copper, Plays a Critical Role in Hydrogen Peroxide-Induced DNA Damage. Free. Radic. Biol. Med..

